# Comprehensive Analyses of Immune Subtypes of Stomach Adenocarcinoma for mRNA Vaccination

**DOI:** 10.3389/fimmu.2022.827506

**Published:** 2022-07-07

**Authors:** Weiqiang You, Jian Ouyang, Zerong Cai, Yufeng Chen, Xiaojian Wu

**Affiliations:** ^1^ Department of Colorectal Surgery, The Sixth Affiliated Hospital, Sun Yat-sen University, Guangzhou, China; ^2^ Guangdong Provincial Key Laboratory of Colorectal and Pelvic Floor Diseases, The Sixth Affiliated Hospital, Sun Yat-sen University, Guangzhou, China; ^3^ The Center for Bioinformatics and Computational Biology, Shanghai Key Laboratory of Regulatory Biology, The Institute of Biomedical Sciences and School of Life Sciences, East China Normal University, Shanghai, China; ^4^ Shanghai-MOST Key Laboratory of Health and Disease Genomics, Institute for Genome and Bioinformatics, Shanghai Institute for Biomedical and Pharmaceutical Technologies, Shanghai, China

**Keywords:** mRNA vaccine, immune subtype, stomach adenocarcinoma, tumor immune microenvironment, immunotherapy

## Abstract

**Background:**

Although messenger RNA (mRNA) vaccines have unique advantages against multiple tumors, mRNA vaccine targets in stomach adenocarcinoma (STAD) remain unknown. The potential effectiveness of mRNA vaccines is closely associated with the tumor immune infiltration microenvironment. The present study aimed to identify tumor antigens of STAD as mRNA vaccine targets and systematically determine immune subtypes (ISs) of STAD that might be suitable for immunotherapy.

**Methods:**

Gene expression profiles and clinical data of patients with gastric cancer were downloaded from The Cancer Genome Atlas (TCGA; n = 409) and the Gene Expression Omnibus (GEO; n = 433), and genomic data were extracted from cBioPortal. Differential gene expression was analyzed using the limma package, genetic alterations were visualized using maftools, and prognosis was analyzed using ToPP. Correlations between gene expression and immune infiltration were calculated using TIMER software, and potential ISs were identified using ConsensusClusterPlus. Functional enrichment was analyzed in clusterProfiler, and r co-expression networks were analyzed using the weighted gene co-expression network analysis (WGCNA) package in R.

**Results:**

Overexpression of the prognostic and highly mutated antigens ADAMTS18, COL10A1, PPEF1, and STRA6 was associated with infiltration by antigen-presenting cells in STAD. Five ISs (IS1–IS5) in STAD with distinct prognoses were developed and validated in TCGA and GEO databases. The tumor mutational burden and molecular and clinical characteristics significantly differed among IS1–IS5. Both IS1 and IS2 were associated with a high mutational burden, massive infiltration by immune cells, especially antigen-presenting cells, and better survival compared with the other subtypes. Both IS4 and IS5 were associated with cold immune infiltration and correlated with advanced pathological stages. We analyzed the immune microenvironments of five subtypes of immune modulators and biomarkers to select suitable populations for mRNA vaccination and established four co-expressed key modules to validate the characteristics of the ISs. Finally, the correlation of these four mRNA vaccine targets with the transcription factors of DC cells, including BATF3, IRF4, IRF8, ZEB2, ID2, KLF4, E2-2, and IKZF1, were explored to reveal the underlying mechanisms.

**Conclusions:**

ADAMTS18, COL10A1, PPEF1, and STRA6 are potential mRNA vaccine candidates for STAD. Patients with IS1 and IS2 are suitable populations for mRNA vaccination immunotherapy.

## Introduction

Gastric cancer (GC), one of the most common malignancies, is a prevalent cause of cancer-related death worldwide, particularly in East Asia (1, 2). Stomach adenocarcinoma (STAD) is the most widespread pathological type of GC, accounting for more than 95% of cases (3). Radical resection followed by adjuvant therapy is the standard treatment for STAD. However, the 5-year survival rate is ~50% despite standard treatment, and STAD recurs or metastasises within 5 years in 70% of patients (4). Immune checkpoint inhibitors (ICIs) exert antitumor effects against several refractory types of human cancer, such as melanoma and non-small cell lung, liver, and renal cell cancers (5–7). However, ICIs have not been similarly effective against STAD, as the objective response rate is favorable for only a few patients with microsatellite instability-high (MSI-H) tumors (8, 9). Therapeutic strategies for STAD are futile; therefore, new functional and efficient approaches to treating STAD are urgently required.

Cancer vaccines can reactivate the immune system to identify and attack tumor cells. They have good prospects for broad clinical application because of their low toxicity, high specificity, and permanent anticancer efficiency. Anti-STAD vaccines have significantly progressed. Tumor-specific T cells combined with chemotherapy can significantly prolong the survival of patients with advanced GC with satisfactory safety and efficiency (10–12). Dendritic cell (DC) vaccines have powerful ability to stimulate the immune system by presenting antigens and activating T cells to release cytokines that eliminate tumors (13). The results of a phase I/II study have suggested that DC vaccines could stimulate immunity and benefit patients with advanced breast, ovarian, and gastric cancers (14). Peptide vaccines are also quite safe but do not confer a significant survival benefit on patients with GC (15). Compared with these types of vaccines, DNA and RNA vaccines are not infectious, have low resistance, are free of contamination, and elicit powerful T-cell responses (16, 17). The DNA vaccines can encode tumor antigens that are recognized by DCs and active T cells to produce tumor immune responses. However, they have the potential risk of integration into the host genome, leading to insertion mutations (18). Major breakthroughs in biotechnology and immunology have driven the recent emergence of messenger RNA (mRNA) vaccines as powerful tools with several advantages for treating human cancers. The safety of mRNA vaccines is favorable because mRNA is non-infectious and non-integrated (19), and they have infinite production potential *via* extensive transcriptional amplification *in vitro* (20). Ultimately, mRNA is easily degraded by cellular RNA enzymes. Moreover, mRNA vaccines can be easily modified to reduce adverse immune responses (21). mRNA vaccines for lung and prostate cancers induce good immune responses and significantly improve prognosis (22, 23). However, mRNA vaccines have not yet been developed for STAD, to the best of our knowledge. In addition, the immune microenvironment of STAD is complex, but immune subtypes (ISs) might contribute to the identification of suitable patients with STAD who could benefit from mRNA vaccination.

We initially screened genes that were abnormally expressed, commonly mutated, and occurred at a high mutation frequency in STAD. We identified four candidate genes that were distinctly associated with a poor prognosis and tumor immune cell infiltration, as potential mRNA vaccine targets. Based on the expression of immune-related genes, we defined and validated five ISs in an independent cohort. These ISs helped predict different outcomes, molecular characteristics, and the immune microenvironment. We then mapped the immune landscape of STAD by analyzing infiltrative immune cells in the tumor microenvironment. Collectively, our results revealed a heterogeneous tumor immune microenvironment (TIME) in patients with STAD; therefore, we propose a new theoretical strategy for treating STAD.

## Materials and Methods

### Data Resources

Gene expression profile and the clinical data for stomach cancer (STAD) patients were separately obtained from The Cancer Genome Atlas (TCGA, https://tcga-data.nci.nih.gov/tcga/) and Gene Expression Omnibus (GEO, https://www.ncbi.nlm.nih.gov/geo/). Genomics data for STAD was downloaded from the cBioPortal data portal (24) (https://www.cbioportal.org/).

### Data Preprocessing

First, normal tissue samples were excluded, and we retained only primary STAD tumor samples. Then, patients without follow-up data were also excluded. Finally, a total of 409 patients in the TCGA cohort and 433 patients in the GEO cohort (GSE84437) were included in this study. In addition, genes with low expression abundance (>80% of samples are 0) were eliminated.

### Differential Gene Expression Analysis and Enrichment Analysis

The differential gene expression analysis for RNA-seq data was performed by the “limma” package (version 3.40.6) in R language. Those genes with FDR < 0.05 and log_2_(FC) > 1 were considered significantly different. Then, all the differential genes were located to chromosomes by the R package “RIdeogram” (version 0.2.2). The enrichment analysis in GO and KEGG pathways were performed by “clusterProfiler” (25) (version 4.2.0) package for all the differential expression genes or gene sets, and adjusted p < 0.05 was considered significantly enriched.

### Genomics Data

The maf file for all the STAD patients in the TCGA cohort was provided by cBioPortal. Then, we used the “maftools” package (version 2.0.16) to analyze and visualize genomic data. In addition, a gene mutation frequency >1% of samples is considered a high-frequency mutation gene.

### Survival Analysis

The univariate analysis for a single gene in the TCGA-STAD cohort was provided by the ToPP database (http://www.biostatistics.online/topp/index.php). The analysis of prognosis in each immune subtype was performed in the “rms” package (version 5.1-3.1) with the Cox proportional hazards model. The log-rank test was used to verify whether there is a significant difference.

### Identification of Immune Subtypes

A total of 2,483 immune-related genes that belong to 17 categories of immune were extracted from the import database (https://www.immport.org/). Then, we intersected these genes with the gene list in TCGA and GEO datasets, and finally, 1,215 genes were retained for subsequent analysis. Consistent clustering provided by “ConsensusClusterPlus” (version 1.48.0) was used to discover the immune subtypes in the TCGA cohort and verified in an independent GEO cohort. The parameter setting of ConsensusClusterPlus includes setting the number of clusters from 2 to 10 and the clustering algorithm to “k-means” using “1 − Pearson correlation” value to calculate the distance, and other settings are the default parameters.

### Correlation Between Gene Expression and Immune Cell Infiltration

TCGA was an important database for analyzing the composition of complex immune cells in tumor microenvironment (26). The correlation between one gene expression and immune infiltrates was calculated in Tumor Immune Estimation Resource (27) (TIMER, https://cistrome.shinyapps.io/timer/) by Spearman correlation analysis. The abundances of six immune infiltrates (B cells, CD4+ T cells, CD8+ T cells, neutrophils, macrophages, and dendritic cells) are estimated by the TIMER algorithm. The single sample gene set enrichment analysis (ssGSEA) (28) analysis was applied to find the significantly enriched immune cell types or related functions. EPIC (29) and McP-Counter (30) were used to estimate the immune cell infiltration from gene expression profiles. One-way ANOVA analysis was used to test the significant differences. Here, we use GSVA (version 1.1.1) package to estimate GSVA enrichment scores for all the KEGG pathway and GO BP terms; then, “limma” package was used to calculate differential pathways or BP terms for the enrichment scores, and adjusted p < 0.05 was considered significantly different.

### Identification of Co-Expression Modules

Core modules and central genes related to STAD were identified through a weighted gene co-expression network analysis (WGCNA) (31) in R. The KEGG pathway enrichment analysis for each module was performed by “clusterProfiler” (version 1.1.1) package, and adjusted p-value <0.05 was considered significantly enriched.

### Statistical Analysis

All the statistical analyses were performed in R language (version 3.6.1). Wilcoxon test was used for the difference test between the two groups, and ANOVA test was used for multiple groups. p < 0.05 was considered significant difference.

## Results

### Identification of Differentially Expressed Genes and High-Frequency Mutant Genes in STAD

We selected differentially expressed genes (DEGs) from The Cancer Genome Atlas (TCGA) of tumor and adjacent normal tissues to uncover potential immune antigens of STAD. The results revealed 1,250 DEGs in STAD, of which 842 and 408 were up- and downregulated, respectively. We also determined their distribution on chromosomes (**Supplementary Figure 1A**). Whether mutations occurred in these differential genes in STAD was determined by analyzing characteristics such as gene mutation classification, mutation type, numbers of single nucleotide variants (SNVs), and the top 10 genes with the highest mutation frequency. Most DEGs had missense mutations, and the most prevalent single-base mutation was C > T. An average of 82 mutations were identified in each gene, and the titan (TTN), mucin 16 (MUC16), low-density lipoprotein receptor-related protein 1B (LRP1B), AT-rich interactive domain-containing protein 1A (ARID1A), spectrin repeat containing nuclear envelope protein 1 (SYNE1), FAT atypical cadherin 4 (FAT4), CUB and sushi multiple domains 3 (CSMD3), piccolo presynaptic cytomatrix protein (PCLO), hemicentin (HMCN1), and zinc finger homeobox 4 (ZFHX4) genes harbored the most mutations (**Supplementary Figure 1B**). The types of mutations in the top 20 DEGs in each STAD sample were mapped (**Supplementary Figure 1C**).

### Identification of Potential Prognostic Markers for STAD

**Figure 1:**
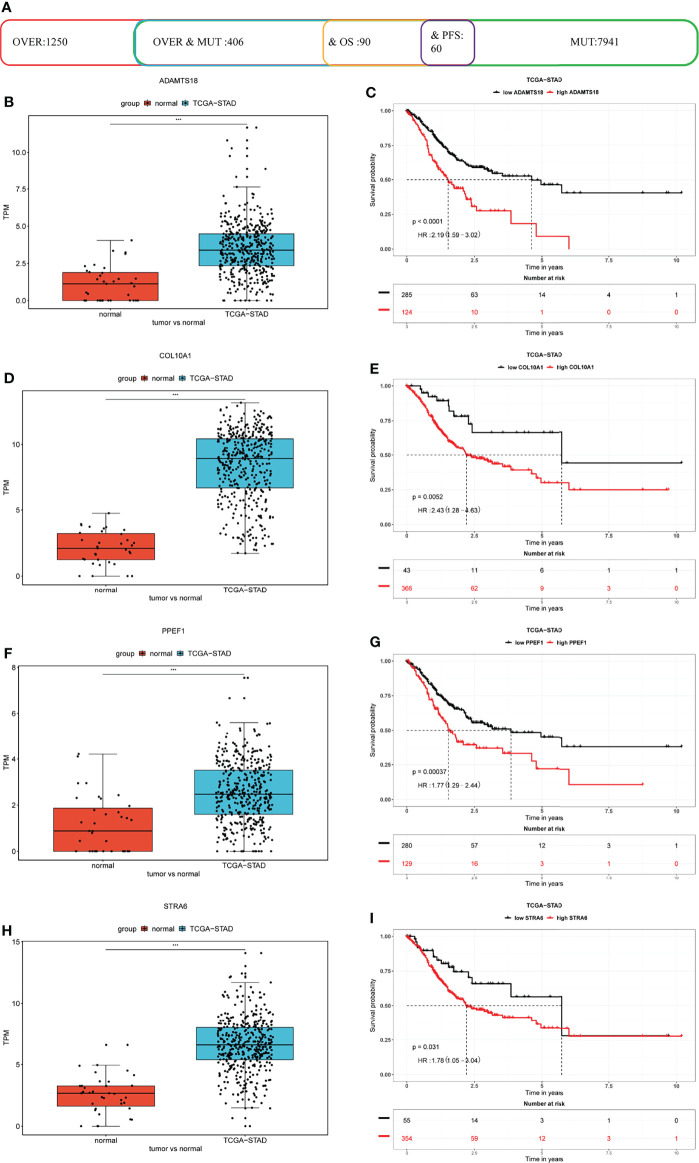
Identification of potential prognostic markers for STAD. **(A)** Statistical data of differentially expressed, high-frequency mutation, and prognosis-related genes in TCGA cohort. **(B, D, F, H)** ADAMTS18, COL10A1, PPEF1, and STRA6 are differentially expressed in STAD compared with that in adjacent normal tissues. **(C, E, G, I)** ADAMTS18, COL10A1, PPEF1, and STRA6 are risk factors for poor prognoses.

We then analyzed 406 high-frequency mutated DEGs in STAD to determine their associations with prognosis and selected 90 and 60 mutated DEGs that were, respectively, related to overall survival (OS) and progression-free survival (PFS) (**Figure 1A**). The results of ToPP prognostic analysis showed that a disintegrin-like and metalloproteinase with thrombospondin type 1 motif 18 (ADAMTS18), collagen type X alpha 1 chain (COL10A1), protein phosphatase with EF-hand domain 1 (PPEF1), and stimulated by retinoic acid 6 (STRA6) were abundantly expressed in STAD; this was associated with a dismal prognosis for patients with STAD (**Figures 1B** **–I**).

### Identification Potential Tumor Antigens Related to Tumor Immune Cell Infiltration

**Figure 2:**
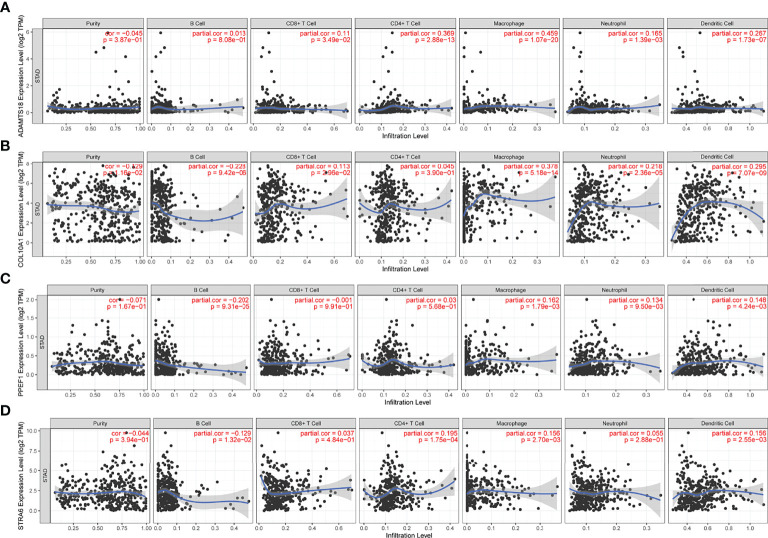
Correlations between gene expression and immune cells. Expression of ADAMTS18 **(A)**, COL10A1 **(B)**, PPEF1 **(C)**, and STRA6 **(D)** correlates with six immune cell types (B cells, CD8^+^ T cells, CD4^+^ T cells, macrophages, neutrophils, and dendritic cells).

To identify potential antigen-associated markers associated with antigen-presenting cells (APCs) in STAD, we assessed correlations between the above prognostic genes and immune cell infiltration. **Figure 2** shows that upregulated ADAMTS18 expression correlates positively with of CD8^+^ T cell (r = 0.11, p < 0.05), CD4^+^ T cell (r = 0.369, p < 0.05), macrophages (r = 0.45, p < 0.05), neutrophil (r = 0.165, p < 0.05), and dendritic cell (DCs; r = 0.267, p < 0.05), but not B-cell infiltration (**Figure 2A**). Upregulated COL10A1 expression was associated positively with infiltration by CD8^+^ T cells (r = 0.113, p < 0.05), macrophages (r = 0.378, p < 0.05), neutrophils (r = 0.218, p < 0.05), and DCs (r = 0.295, p < 0.05), and negatively with B cells (r = −0.228, p < 0.05) but not CD4^+^ T cells (**Figure 2B**). Upregulated PPEF1 expression was related to extensive infiltration by macrophages (r = 0.162, p < 0.05), neutrophils (r = 0.134, p < 0.05), and DCs (r = 0.148, p < 0.05), and scant B cells in the microenvironment (r = −0.202, p < 0.05; **Figure 2C**). Moreover, STRA6 expression was associated with abundant infiltration by CD4^+^ T cells (r = 0.195, p < 0.05), macrophages (r = 0.156, p < 0.05), and DCs (r = 0.156, p < 0.05) but few B cells (r = −0.129, p < 0.05; **Figure 2D**). Collectively, these results showed that upregulated ADAMTS18, COL10A1, PPEF1, and STRA6 expression was significantly associated with increased tumor microenvironment infiltration by macrophages and DCs (**Figures 2A** **–D**). These findings indicated that DCs can function as APCs by recognizing and presenting tumor antigens to T cells to activate the immune response (32).

### Development and Validation of Immune Subtypes of STAD

**Figure 3:**
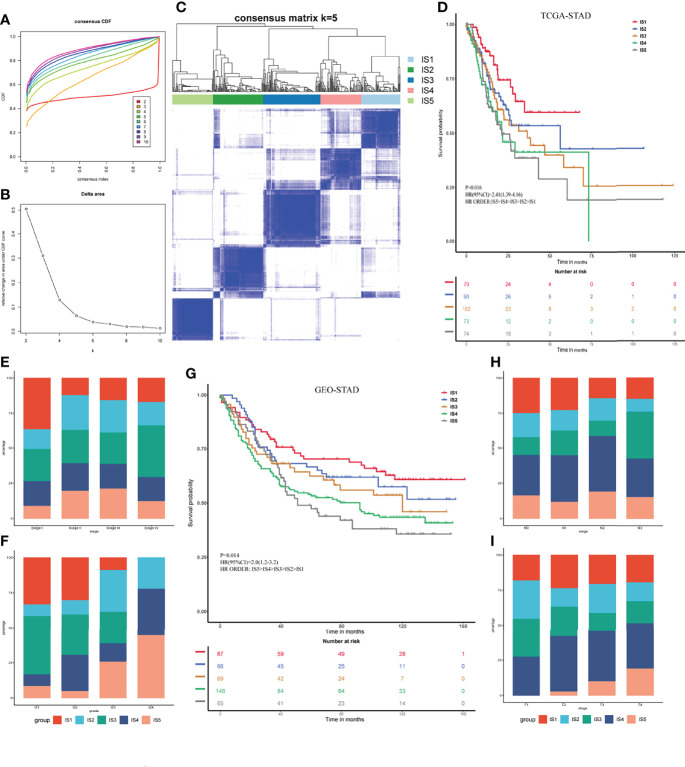
Identification of immune subtypes for STAD. **(A)** Consensus cumulative distribution function for K = 2–10. **(B)** Delta area for K = 2–10. **(C)** Immune subtypes (IS1–IS5) in TCGA cohort at K = 5. **(D)** Prognosis associated with five immune subtypes in TCGA cohort. **(E, F)** Distribution of IS1–IS5 across gastric cancer stages **(E)** and grades **(F)** in TCGA cohort. **(G)** Prognosis associated with five immune subtypes in GEO cohort. **(H, I)** Distribution of IS1–IS5 across gastric cancer N **(H)** and T **(I)** stages in GEO cohort.

The TIME plays a vital role in tumor invasion and metastasis and predicts the efficiency of vaccines (33). Considering that TIME is heterogeneous among patients, identifying ISs is essential to selecting appropriate populations that would benefit from mRNA vaccine therapy. Here, we screened 1,215 immune-related genes for sub-classification in the STAD cohorts downloaded from TCGA and GEO. The genes were classified using the ConsensusClusterPlus function. The classification value k = 5 was selected according to the consensus CDF and delta area (**Figures 3A** **, B**). Thus, the patients with STAD in TCGA cohort were clustered into the subtypes IS1–IS5 (**Figure 3C**). The results of survival analyses suggested that patients with IS1 and IS2 would have a favorable prognosis, whereas those with IS4 and IS5 would not (p = 0.016; **Figure 3D**). We then analyzed the proportion of ISs at different various pathological stages and neoplasm histological grades. **Figure 3E** shows that patients with stage I had a large proportion of IS1, whereas those with stages II and III had decreased IS1 and increased IS5. Most grade 1 and 2 tumors were classified as IS1–IS3, whereas most grade 3 samples were classified as IS4 and IS5 (**Figure 3F**). We verified these classifications of the five ISs in the GEO STAD cohort (p = 0.014; **Figure 3G**). We analyzed the proportions of these five ISs in the T and N stages and found high proportions of IS1 and IS2 in N0 and N1, whereas IS3–IS5 predominated in N2 and N3 (**Figure 3H**). Similarly, IS4 and IS5 proportions were relatively low in T1 and T2, and IS5 was absent in T1. However, the proportions of IS4 and IS5 were significantly increased in patients with T3 and T4 (**Figure 3I**). These results indicated that the ISs are significantly associated with STAD prognosis. The results also overlapped with contemporary methods of clinical staging.

Meanwhile, the GSVA analysis was performed in BP and KEGG pathway for all the immune-reactivity-related genes. The results showed that these subtypes had significant differences in the regulation of proteolysis, cytosolic calcium ion concentration, divalent inorganic cation homeostasis, and calcium ion transport for biological process (BP, **Supplementary Figure 3**). The KEGG analysis suggested that changed pathways were enriched in calcium signaling, intestinal immune network for IgA production, systemic lupus erythematosus, antigen processing and presentation, dendritic cell antigen processing and presentation, and regulation of immune system process (**Supplementary Figure S3**).

### Analysis of Tumor Mutational Burden in Immune Subtypes

**Figure 4:**
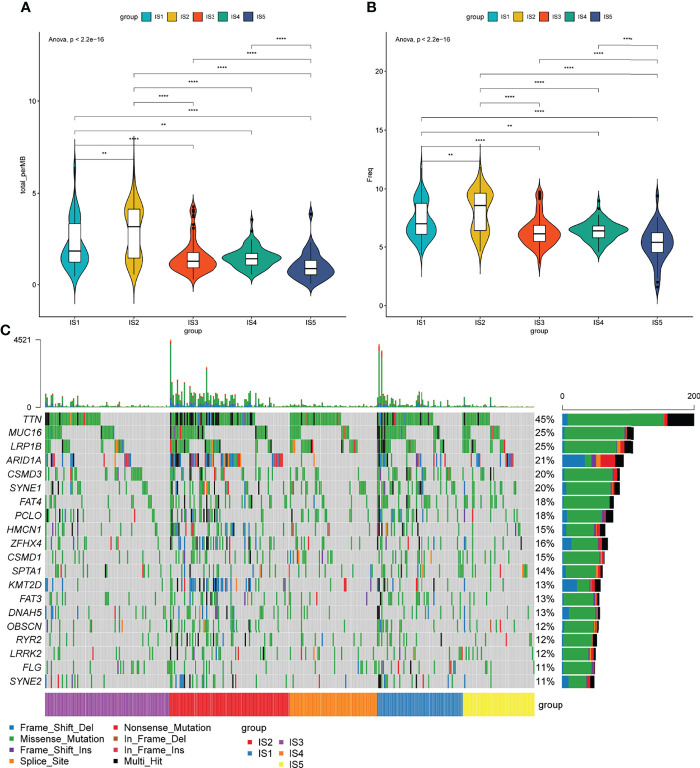
Association between immune subtypes and tumor mutational burden. **(A)** Tumor mutational burden, **(B)** number of mutated genes, and **(C)** top 20 high-frequency mutation genes in IS1–IS5.

Tumor mutational burden (TMB) is closely associated with cancer immunotherapy (34). Tumors with high TMB have an elevated neoantigen load and a durable immune response (35). Therefore, the TMB levels of each immune subgroup in TCGA were evaluated. The TMB levels of IS1 and IS2 were significantly higher than those of IS4 and IS5 (p < 0.05, **Figure 4A**). Notably, trends in the numbers of mutated genes were similar between IS1 and IS2 (p < 0.05, **Figure 4B**). The top 20 mutated genes TTN, MUC16, LRP1B, ARID1A, CSMD3, SYNE1, FAT4, PCLO, HMCN1, ZFHX4, CSMD1, SPTA1, KMT2D, FAT3, DNAH5, OBSCN, RYR2, LRRK2, FLG, and SYNE2 were identified in patients with STAD (**Figure 4C**). Almost 50% of the patients had TTN mutations, whereas 25% had mutations in MUC16 and LRP1B (**Figure 4C**). These results indicated that ISs could predict TMB levels and somatic mutation rates in patients with STAD and that patients with IS1 and IS2 might respond positively to mRNA vaccines.

### Association Between Immune Modulators and Immune Subtypes in STAD

**Figure 5:**
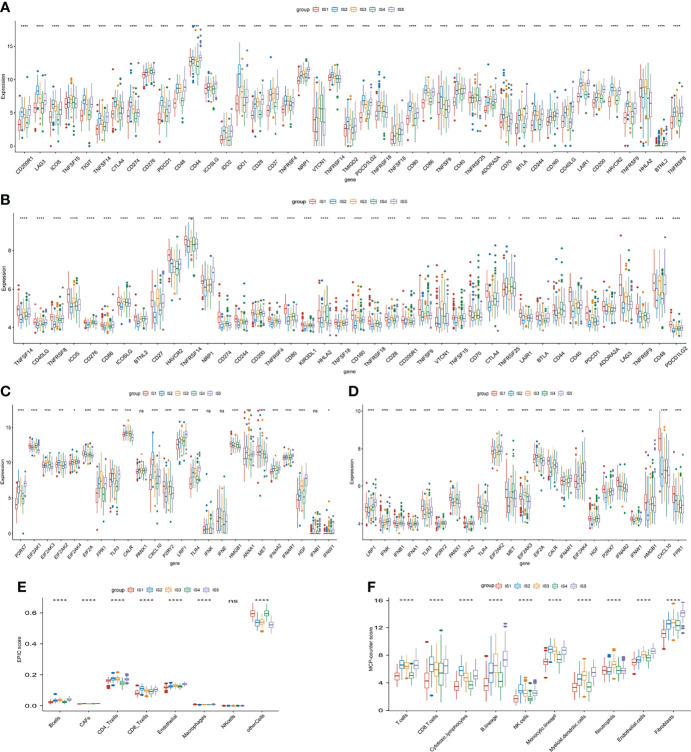
Immune checkpoints (ICPs), immunogenic cell death (ICD) regulators, and immune infiltration related to immune subtypes. **(A, B)** Expression of 43 ICP-related genes significantly differs in all subgroups in TCGA **(A)** and GEO **(B)** cohorts. **(C, D)** Significantly different expression of ICD-related genes in TCGA **(C)**, n = 20) and GEO cohort **(D)**, n = 23). **(E, F)** Differences of immune-infiltrating cells among five subgroups performed by EPIC **(E)** and McP-Counter **(F)**. *p < 0.05, **p < 0.01, ***p < 0.001, and ****p < 0.0001.

Considering the significance of immune checkpoints (ICPs) and immunogenic cell death (ICD) regulators in cancer immunity (36), we analyzed the expression of the various subtypes. The expression of 43 ICP-related genes in TCGA and GEO datasets significantly differed among the five ISs (**Figures 5A** **, B**). For instance, lymphocyte activation gene 3 (LAG3), inducible T cell costimulator (ICOS), T-cell immunoreceptor with immunoglobulin and ITIM domains (TIGIT), tumor necrosis factor superfamily member 14 (TNFSF14), cytotoxic T-lymphocyte–associated antigen 4 (CTLA4), cluster of differentiation 274 (CD274), programmed cell death protein 1 (PDCD1), indoleamine 2,3-dioxygenase (IDO1), transmembrane and immunoglobulin domain-containing 2 (TMIGD2), TNF superfamily member 9 (TNFSF9), cluster of differentiation (CD244), and hepatitis A virus cellular receptor 2 (HAVCR2) were significantly overexpressed in IS2 tumors in TCGA cohort and in IS1 tumors in the GEO cohort. The expression of 24 ICD-related genes in TCGA cohort and 23 ICD-related genes in the GEO cohort was also significantly different among the five subtypes (**Figures 5C** **, D**). The expression of purinergic receptor P2X 7 (P2RX7), toll-like receptor (TLR3), C-X-C motif chemokine ligand 10 (CXCL10), toll-like receptor 4 (TLR4), and MNNG-HOS transforming (MET) was significantly high in IS2 and IS1 tumors in TCGA and GEO cohorts, respectively. These results indicated that ISs have important guiding significance in immunotherapy. Furthermore, EPIC and McP-Counter methods were performed to analyze the immune cell groups in the five subgroups. The EPIC results showed that there were significant differences in B cells, CD4^+^ T cells, CD8^+^ T cells, and NK cells (**Figure 5E**), which was consistent with the McP-Counter results (**Figure 5F**).

### The Relationship Between Serum Tumor Markers and Immune Subtypes in STAD

Serum tumor markers play vital roles in the early diagnosis and prognosis of cancer. Alpha-fetoprotein (AFP), carbohydrate antigen 125 (CA125), carbohydrate antigen 19-9 (CA19-9), and carcinoembryonic antigen (CEA) are the most common tumor markers in clinical practice. Here, we analyzed the expression of CEA, CA125, and CA19-9 in various STAD ISs in TCGA and GEO cohorts. The expression of these tumor markers differed among the subtypes in the two cohorts. The expression of CEA was significantly higher and lower in the IS1 and IS5 subtypes, respectively, in TCGA and GEO cohorts (p < 0.05, **Supplementary Figures 2A, B**). Significantly more CA125 was expressed in the IS2 than in the other subtypes in TCGA cohort (p < 0.05; **Figure S2C**) and in IS1 tumors in the GEO cohort than in the other four subtypes (p < 0.05, **Supplementary Figure 2D**). The expression of CA19-9 was significantly increased in the IS5 subgroup compared with the other subgroups in both TCGA and GEO cohorts (p < 0.05, **Supplementary Figures 2E, F**). These results indicated that ISs were associated with molecular markers and had favorable guiding significance for prognosis.

### The Immune Cell Function in Immune Subtypes

**Figure 6:**
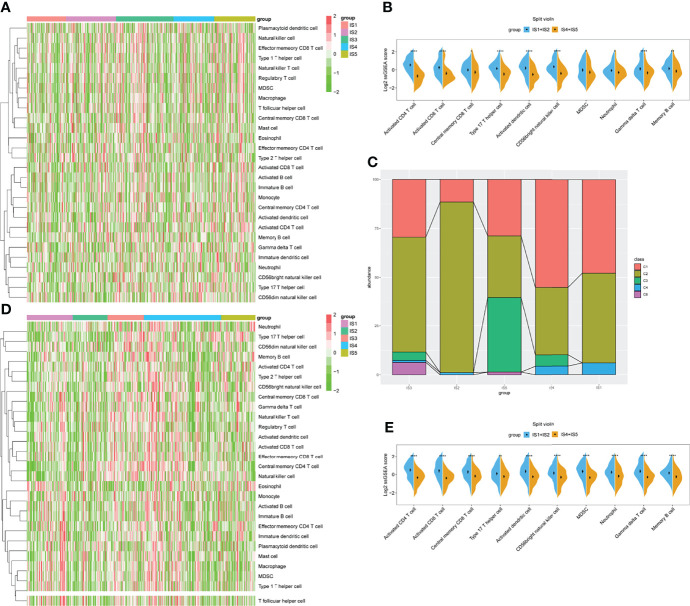
Identification of immune cells in immune subtypes (ISs) for STAD. **(A)** Heatmap of ssGSEA showing scores for types of immune cells with IS1–IS5 in TCGA cohort. **(B)** Immune cell types significantly differ between IS1+IS2 and IS4+IS5 groups in TCGA cohort. **(C)** Distribution of IS1–IS5 across C1–C6 in TCGA cohort. **(D)** Heatmap of ssGSEA showing scores for immune cell types with IS1–IS5 in GEO cohort. **(E)** Immune cell types significantly differ between IS1+IS2 and IS4+IS5 groups in GEO cohort. *p < 0.05, **p < 0.01, ***p < 0.001, and ****p < 0.0001.

Immune cell activity and function are critical for the immune response (37). We evaluated the IS enrichment in various immune cells using single sample gene set enrichment analysis (ssGSEA) to characterize immune cell components using 28 reported signatures. The ssGSEA score for each immune cell in TCGA cohort was then applied to sample clustering. The IS1 and IS2 subgroups and the IS4 and IS5 subgroups had similar scores for several immune cell types (**Figure 6A**). Among them, the IS1 and IS2 subgroups contained significantly more activated CD4 T cells, CD8 T cells, and DCs, central memory CD8 T cells, type 17 T helper cells, CD56 bright natural killer cells, myeloid-derived suppressor cells (MDSCs), neutrophils, gamma delta T cells, and memory B cells compared with the other subtypes (p < 0.05, **Figure 6B**). Thus, IS1 and IS2 were immunologically “hot,” while IS4 and IS5 were immunological “cold” phenotypes. The findings of the GEO cohort were similar (p < 0.05; **Figures 6D** **, E**). Subsequently, we screened the differentially expressed genes between the two groups (IS1 + IS2 vs. IS4 + IS5). The GO analysis showed that these genes were mainly enriched in the regulation of neuron projection development, neuron projection morphogenesis, plasma membrane bounded cell projection morphogenesis, cell part morphogenesis, and cell projection morphogenesis for BP (**Supplementary Figure S4A**), and axon, supramolecular complex, cell body, cell–cell junction, and neuron projection for cellular component (CC, **Supplementary Figure 4B**). The KEGG analysis demonstrated that NOD-like receptor signaling pathway, IL-17 signaling pathway, cell cycle, rheumatoid arthritis, ECM–receptor interaction, and TNF signaling pathway were enriched (**Supplementary Figure 4C**). The changes in these pathways were closely related to the immune microenvironment and tumor progression.

We also compared the accuracy of our definition of the five ISs with six previously defined immune clusters (C1–C6) (38). The results showed that IS1 and IS2 considerably overlapped with the C1 and C2 subtypes. Meanwhile, IS5 seemed much similar to the C3 IS, and most samples with the C6 subtype were classified into our IS3 subtype (**Figure 6C**).

### Co-Expression Network Analysis of Immune-Related Genes and Screening of Key Modules

**Figure 7:**
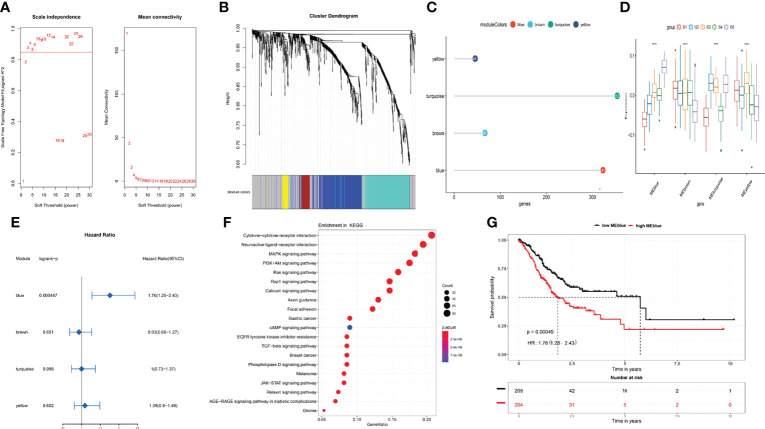
Co-expression network analysis of immune-related genes in STAD. **(A)** Selection of soft threshold for power. **(B)** Co-expression network modules and **(C)** number of immune-related genes in each module. **(D)** Enrichment scores for each module in IS1–IS5 immune subtypes. **(E)** Prognosis for each module in TCGA cohort. **(F)** Blue module, KEGG pathway enrichment. **(G)** Overall survival curves differ between low and high blue modules. *p < 0.05, **p < 0.01, ***p < 0.001, and ****p < 0.0001.

Immune-related gene co-expression networks were analyzed using the blockwiseModules function in weighted gene co-expression network analysis (WGCNA) with the parameter settings minModuleSize = 20, mergeCutHeight = 0.25, and others with default settings. The power for blockwise Modules was automatically selected by the comprehensive consideration of scale independence and mean connectivity. We set the power to 3 for module discovery (**Figure 7A**) and identified four clustered co-expression modules (**Figures 7B** **, C**). We calculated the enrichment scores of the five ISs in each module. Tumors with IS5 had the highest score in blue and turquoise modules, whereas those with IS1 had the lowest (p < 0.05, **Figure 7D**). Survival analyses showed that the blue module was a risk factor for the prognosis of STAD [hazard ratio (HR), 1.76; 95% confidence interval (CI), 1.28–2.43; p < 0.05; **Figures 7E** **, G**), whereas other modules did not significantly affect prognosis (**Figure 7E**). Furthermore, genes in the blue module were the most significantly enriched in cytokine–cytokine receptor interaction, neuroactive ligand–receptor interaction, mitogen-activated protein kinase (MAPK) signaling pathway, PI3K-Akt signaling pathway, Ras signaling pathway, and others. All enriched pathways were closely associated with tumor occurrence and development (**Figure 7F**).

### The Potential Mechanisms of the Four Targets for mRNA Vaccine

**Figure 8:**
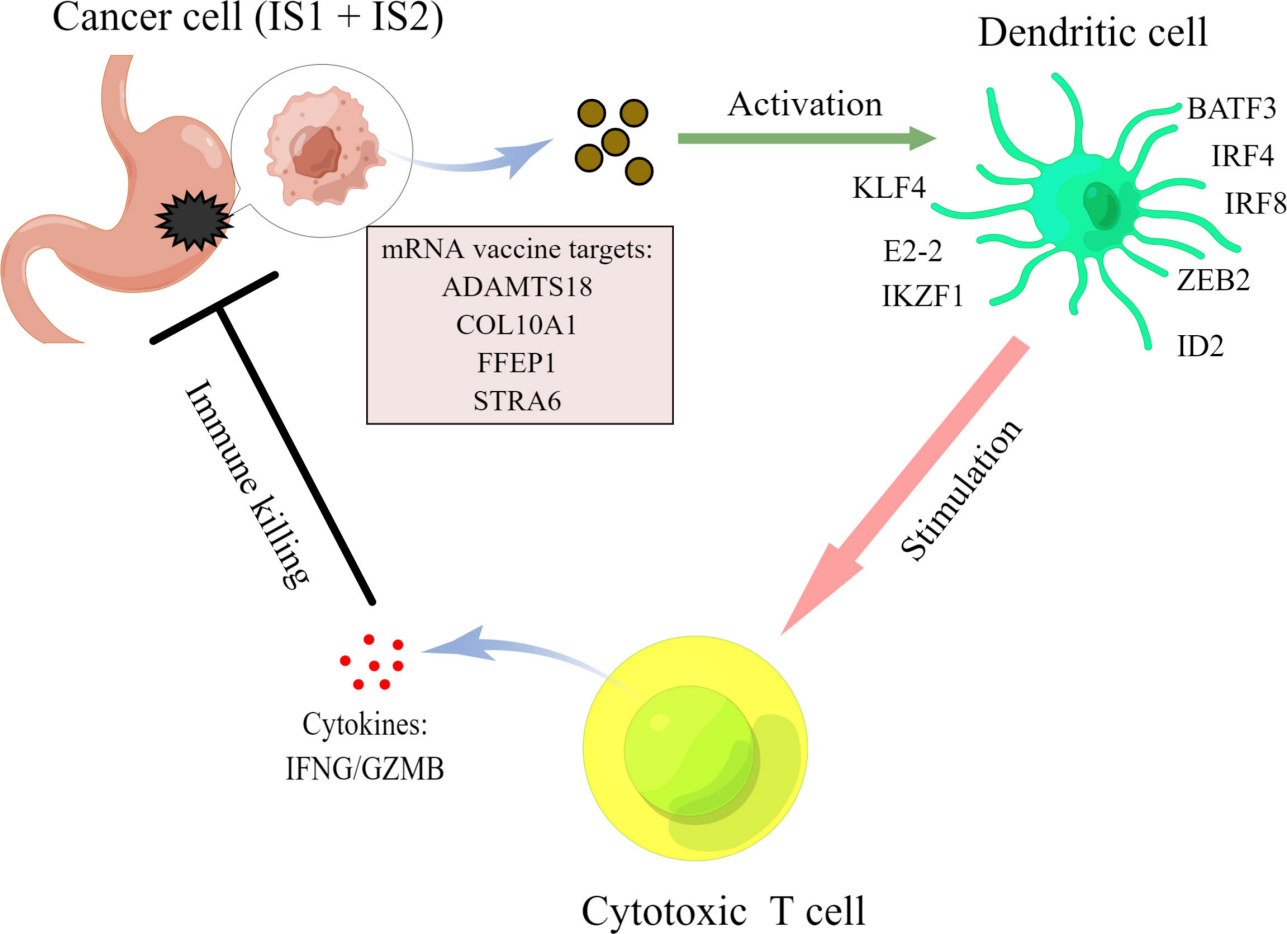
A brief diagrammatic figure to summarize our findings.

Our results suggested that the four mRNA vaccine targets, namely, ADAMTS18, COL10A1, PPEF1, and STRA6, were positively correlated with the infiltration of dendritic cell, thereby activating cytotoxic T cells against tumor cells. To explore the potential mechanisms, we analyzed the relationships between these four targets and transcription factors that regulate dendritic cell function, including BATF3, IRF4, IRF8, ZEB2, ID2, KLF4, E2-2, and IKZF1 (39, 40). As shown in **Table 1**, ADAMTS18 was positively correlated with the expression of BATF3 (r = 0.223, p < 0.05), IRF4 (r = 0.166, p < 0.05), ZEB2 (r = 0.488, p < 0.05), ID2 (r = 0.150, p < 0.05), E2-2 (r = 0.558, p < 0.05), and IKZF1 (r = 0.237, p < 0.05). COL10A1 was associated with the expression of BATF3 (r = 0.293, p < 0.05), ZEB2 (r = 0.360, p < 0.05), and E2-2 (r = 0.300, p < 0.05). PPEF1 was significantly expressed with the level of BATF3 (r = 0.360, p < 0.05), ZEB2 (r = 0.122, p < 0.05), and E2-2 (r = 0.112, p < 0.05). Similarly, STRA6 was also correlated to the expression of BATF3 (r = 0.194, p < 0.05), ZEB2 (r = 0.148, p < 0.05), ID2 (r = 0.104, p < 0.05), and E2-2 (r = 0.210, p < 0.05). Additionally, these four targets were negatively associated with the expression of KLF4 (p < 0.05, **Table 1**). KLF4 regulated a subtype of DC cells that was responsible for CD4 cell differentiation rather than antigen presentation (41). Therefore, the mechanisms of these four mRNA vaccine targets may be closely related to the transcription factors of DC cells (**Table 1**). Our findings were summarized by a brief diagrammatic figure (**Figure 8**).

**Table 1:** The correlation of four mRNA vaccine targets with biomarkers of dendritic cell in TCGA cohort.

Variables	ADAMTS18		COL10A1		PPEF1		STRA6	
	Rho	p	Rho	p	Rho	p	Rho	p
BATF3	0.223	4.55E−06	0.293	1.21E−09	0.200	4.18E−05	0.194	6.95E−05
IRF4	0.166	6.88E−04	−0.003	9.46E−01	−0.036	4.63E-01	0.052	2.90E−−01
IRF8	−0.028	5.64E−01	0.021	6.73E−01	0.041	4.06E−01	−0.070	1.54E−01
ZEB2	0.488	3.64E−26	0.360	3.74E−14	0.122	1.31E−02	0.148	2.53E−03
ID2	0.150	2.23E−03	0.082	9.58E-02	0.086	8.11E−02	0.104	3.43E−02
KLF4	−0.101	4.05E−02	−0.230	2.09E−06	-0.136	5.44E−03	−0.261	7.11E−08
E2−2	0.558	2.63E−35	0.300	4.39E−10	0.112	2.19E−02	0.210	1.68E−05
IKZF1	0.237	1.04E−06	0.070	1.55E−01	0.024	6.29E−01	0.082	9.64E−02

## Discussion

The safety and efficacy of mRNA vaccines have been confirmed in lung cancer (23), breast cancer (42), and melanoma (43). However, these vaccines cannot efficiently treat STAD. Therefore, we systematically analyzed the types and profiles of mutated genes that are prevalent in STAD. Our results suggested that most STADs have missense mutations and that almost 50% of patients have TTN mutations, which were consistent with recently published findings (44). Among mutated and overexpressed genes, ADAMTS18, COL10A1, PPEF1, and STRA6 in STAD were associated with dismal OS and recurrence-free survival; these genes might be good targets for mRNA vaccines. Their expression was associated with tumor infiltration by immune cells, such as macrophages, neutrophils, B cells, CD8^+^ T cells, CD4^+^ T cells, and DCs. The upregulation of four candidate genes notably correlated positively with DC infiltration. Since the antigen encoded by the mRNA vaccine needs to be presented by DCs (45), our results indicated that these four mRNA vaccine targets should be effective against STAD. Moreover, our findings agree with those of several published studies. Mutations in ADAMTS18 play vital roles in promoting cell invasion and metastasis in melanoma (46), and upregulated ADAMTS18 expression predicts poor survival for patients with STAD (47). More COL10A1 is expressed in GC than in normal tissues, and this is distinctly related to T stage and lymph node metastasis (48). In addition, COL10A1 is a carcinogenic marker involving the epithelial-to-mesenchymal transition in GC, which is regulated by the transforming growth factor beta1 (TGF-β1)-SRY-box transcription factor 9 (SOX9) axis (49). Along with being an unfavorable prognostic marker, PPEF1 is negatively associated with OS and metastasis-free survival of breast cancer (50). Lin et al. found that STRA6, targeted by miR-873, exerts tumorigenic functions in GC cells and that STRA6 knockdown inhibits cell proliferation and metastasis by disrupting the Wnt/β-catenin signaling pathway (51). Moreover, STRA6 polymorphisms are associated with epidermal growth factor receptor (EGFR) mutations and might be therapeutic targets for patients with non-small cell lung cancer (52). Taken together, our results elucidated the complex functions of these four candidates in STAD and provided essential theoretical evidence for the development of STAD mRNA vaccines. Nevertheless, these results should be validated by basic experiments and clinical trials in the future.

The TIME contains infiltrative immune cells; it can also determine antitumor effects and predict the efficiency of mRNA vaccines (53). A comprehensive analysis of the TIME of STAD could identify populations that might benefit from mRNA vaccines. Therefore, five ISs were developed based on the expression of immune-related genes. Our results suggested that IS1 and IS2 were associated with better prognosis than the other three subtypes, which was validated in GSE84437, an independent STAD cohort. ISs with a dismal prognosis also tended to associate with advanced pathological stage, tumor grade, and T stage, which was compatible with our clinical findings. The molecular and cellular characteristics of the five ISs were investigated. Immune cell infiltration was significantly different among the five ISs. The TMB is crucial for evaluating the effects of immunotherapy and immune responses in cancer in humans (54, 55). We speculate that mRNA vaccines would be most effective in IS1 and IS2 owing to a higher TMB and somatic mutation frequency. Moreover, the expression of ICP biomarkers, including PDCD1, LAG3, cluster of differentiation 60 (CD160), and CTLA4, was upregulated in IS2 and IS1 in TCGA and GEO cohorts, respectively. This suggested that ICI therapies combined with mRNA vaccines might attain better curative efficiency. Serum tumor markers, including CEA, CA19-9, and CA125, are classic prognostic factors of STAD (56). CEA and CA19-9 were overexpressed in IS4 and IS5 than in the other three subtypes, verifying that IS4 and IS5 are associated with dismal OS. Notably, the HLA subtype was not analyzed in our research, as HLA played a vital role in immunotherapy of multiple tumors but was not strongly correlated with vaccines. The loss of HLA-I was related to initial resistance and secondary immune escape (57).

Considering the microenvironmental heterogeneity of each IS, we analyzed the immune cell infiltration in detail. The ssGSEA results revealed elevated scores for activated CD4 T cells, CD8 T cells, DCs, and CD56 bright natural killer cells in IS1. Notably, IS1 combined with IS2 in TCGA and GEO cohorts had remarkably more infiltration by immune cells, especially DCs, indicating that patients with IS1 and IS2 might have a favorable immune response after mRNA vaccination. Consistent with previous findings, IS4 and IS5 were associated with low infiltration of activated CD4 T cells, gamma delta T cells, CD56 bright natural killer cells, and activated DCs, indicating the absence of immune cells in the tumor microenvironments of IS4 and IS5. This also explains why the prognosis for IS4 and IS5 was the poorest among the five subtypes. Besides, KEGG analysis showed that the changed pathways were strongly associated with the immune microenvironment and tumor progression. Subsequently, we classified STAD into blue, brown, turquoise, and yellow groups using WGCNA. The results indicated that IS1 and IS2 were associated with less “blue” expression, whereas IS4 and IS5 expressed relatively more “blue.” Additionally, blue was a risk factor for OS, and high blue expression indicated a dire prognosis. Thus, these results agreed with our previous conclusions and further confirmed the accuracy of our immunotyping. The results of the KEGG enrichment analysis revealed that the MAPK and PI3K-Akt signaling pathways that correlate with tumorigenicity, invasion, and metastasis of STAD were involved in the “blue” module (58, 59).

## Data Availability Statement

The datasets presented in this study can be found in online repositories. The names of the repository/repositories and accession number(s) can be found in the article/**Supplementary Material**.

## Author Contributions

XW designed this study. WY, JO, ZC, and YC performed bioinformatic analyses. WY collected the results and wrote the manuscript. JO revised the manuscript. All authors have read the final version of this manuscript.

## Funding

This study was supported by the National Key R&D Program of China (No. 2017YFC1308800), National Key Clinical Discipline, National Natural Science Foundation of China (Nos. 81972212 and 82003197), Guangdong Natural Science Foundation (No. 2019A1515010063), Science and Technology Planning Project of Guangdong Province, China (No. 2021A0505030028), Science and Technology Planning Project of Guangzhou City (No. 202102020186), Postdoctoral Fund project of The Sixth Affiliated Hospital, Sun Yat-sen University (No. R20210217202102987), Youth Fund for Basic and Applied Basic Research of Guangdong Province (No. 2021A1515111128), and Shanghai Municipal Health Commission and Collaborative Innovation Cluster Project (No. 2019CXJQ02).

## Conflict of Interest

The authors declare that the research was conducted in the absence of any commercial or financial relationships that could be construed as a potential conflict of interest.

## Publisher’s Note

All claims expressed in this article are solely those of the authors and do not necessarily represent those of their affiliated organizations, or those of the publisher, the editors and the reviewers. Any product that may be evaluated in this article, or claim that may be made by its manufacturer, is not guaranteed or endorsed by the publisher.

## Supplementary Material

Supplementary Figure 1 Identification of potential biomarkers of STAD in TCGA cohort. **(A)** Distribution of differentially upregulated (red) and downregulated (blue) genes in STAD. **(B)** Summary of mutations in STAD. **(C)** The most frequently mutated genes in STAD.

Supplementary Figure 2 Relationships between tumor markers and immune subtypes. Expression of carcinoembryonic antigen (CEA) in IS1–IS5 in TCGA **(A)** and GEO **(B)** cohorts. Expression of carbohydrate antigen 125 (CA125) in IS1–IS5 in TCGA **(C)** and GEO **(D)** cohorts. Expression of carbohydrate antigen 19-9 (CA19-9) in IS1–IS5 in TCGA **(E)** and GEO **(F)** cohorts. *p < 0.05, **p < 0.01, ***p < 0.001, and ****p < 0.0001.

Supplementary Figure 3 The GSVA analysis was performed in BP **(A)** and KEGG pathway **(B)** for the immune reactivity related genes.

Supplementary Figure 4 The differentially expressed genes between the two groups (IS1 + IS2 vs. IS4 + IS5) by GO **(A, B)** and KEGG pathway **(C)**.
