# Supplementary material for: Comprehensive Analyses of Immune Subtypes of Stomach Adenocarcinoma for mRNA Vaccination
Source: Front Immunol. 2022 Jul 7;13:827506. doi: 10.3389/fimmu.2022.827506 (PMC9300892; doi:10.3389/fimmu.2022.827506)
Supplement: Supplementary Figure 1 — Identification of potential biomarkers of STAD in TCGA cohort. (A) Distribution of differentially upregulated (red) and downregulated (blue) genes in STAD. (B) Summary of mutations in STAD. (C) The most frequently mutated genes in STAD. [file DataSheet_1.docx]

Supplementary Material

## Supplementary Figures


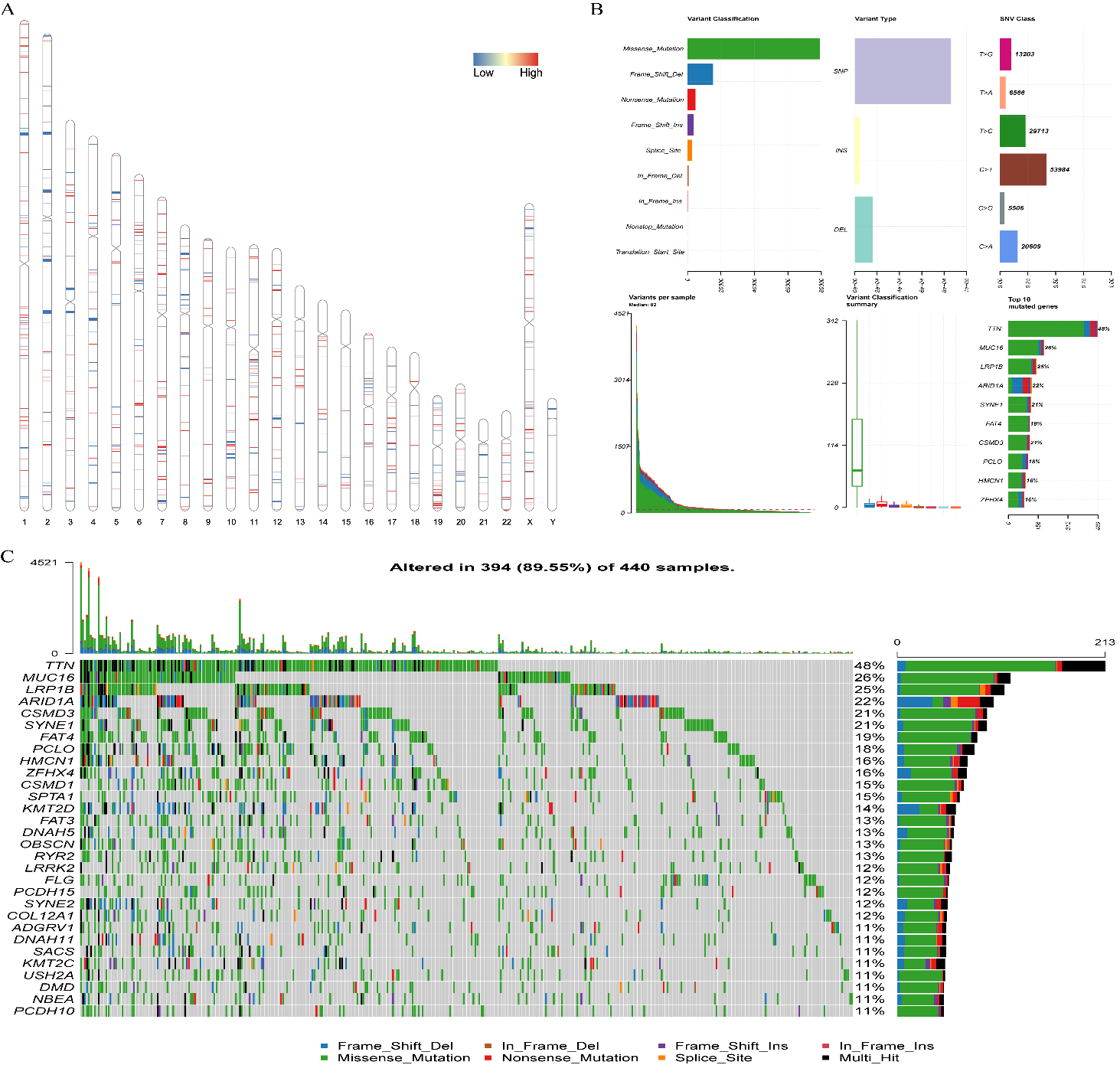


**Supplementary Figure 1.** Identification of potential biomarkers of STAD in TCGA cohort. (A) Distribution of differential genes on chromosomes. Red represents genes that were up-regulated in STAD, and blue represents genes that were down-regulated in STAD. (B) Summary of mutation in STAD. (C) The most frequently mutated gene in STAD.


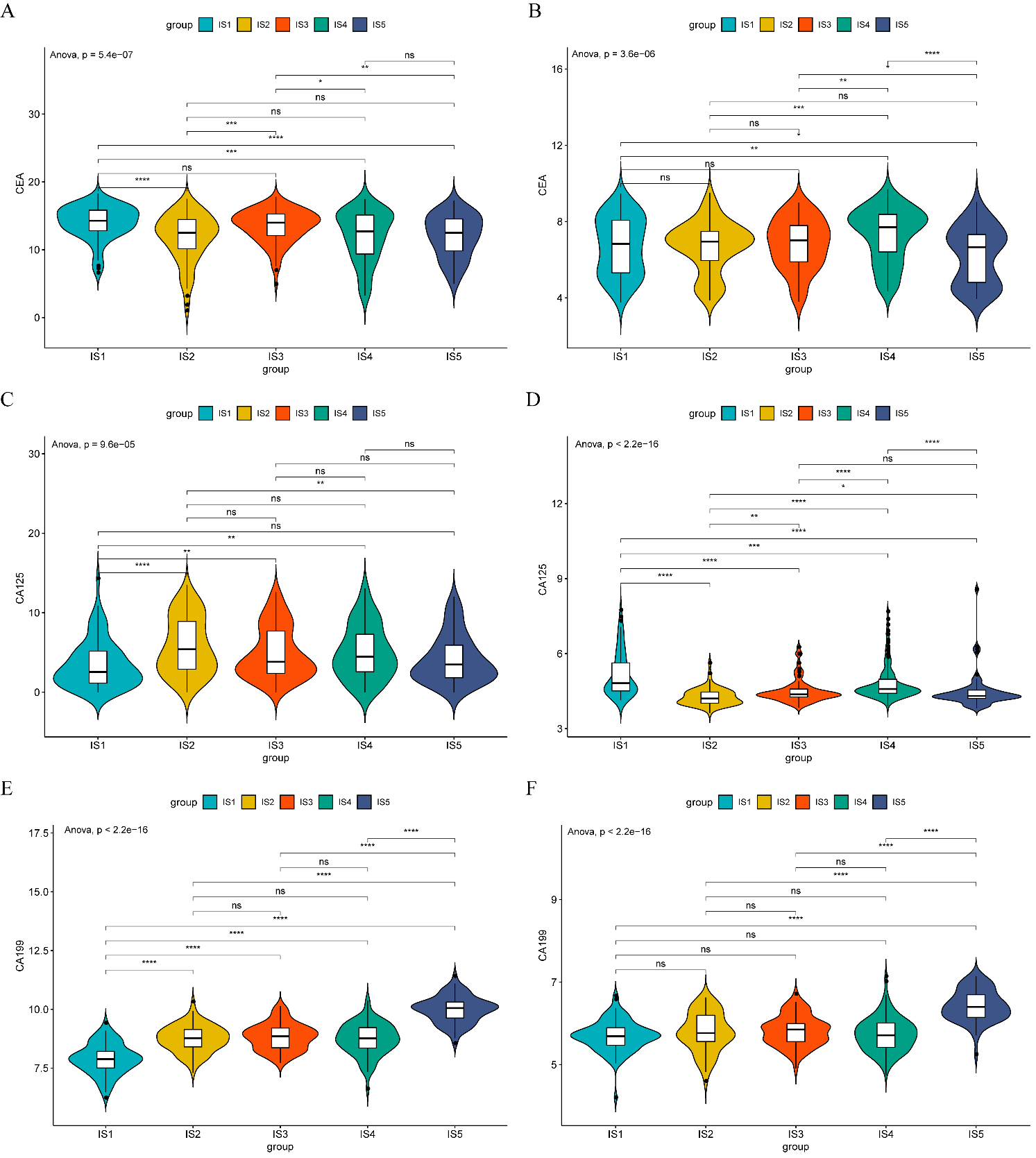


**Supplementary Figure 2.** Relationship between tumor markers and immune subtypes. (A-B) Expression of carcinoembryonic antigen (CEA) in IS1-IS5 in TCGA cohort (A) and GEO cohort (B). (C-D) Expression of carbohydrate antigen 125 (CA125) in IS1-IS5 in TCGA cohort (C) and GEO cohort (D). (E-F) Expression of carbohydrate antigen 19-9 (CA19-9) in IS1-IS5 in TCGA cohort (E) and GEO cohort (F). * p < 0.05, ** p < 0.01, *** p < 0.001, and **** p < 0.0001.


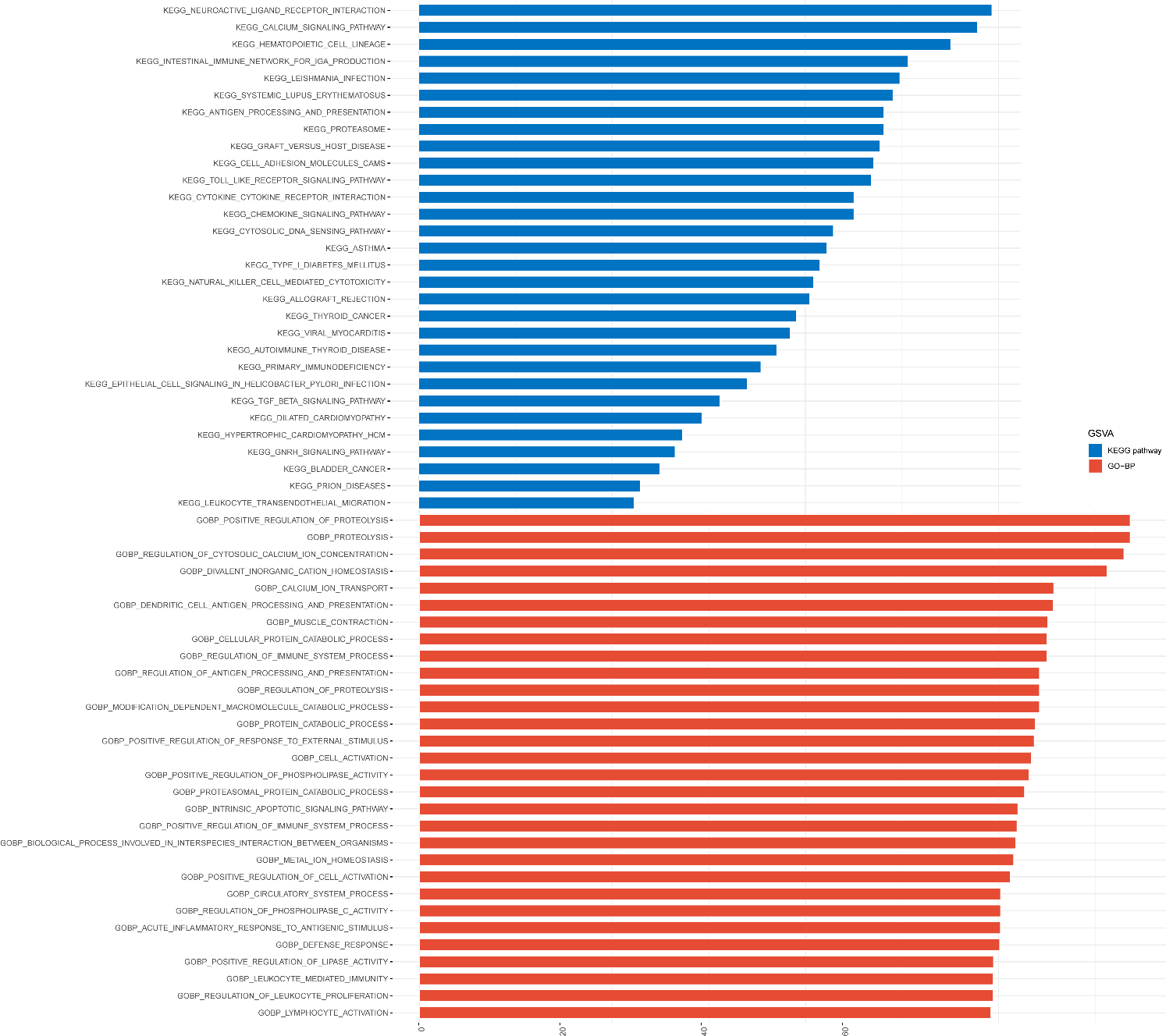


**Supplementary figure 3.** The GSVA analysis was performed in BP (A) and KEGG pathway (B) for the immune reactivity related genes.


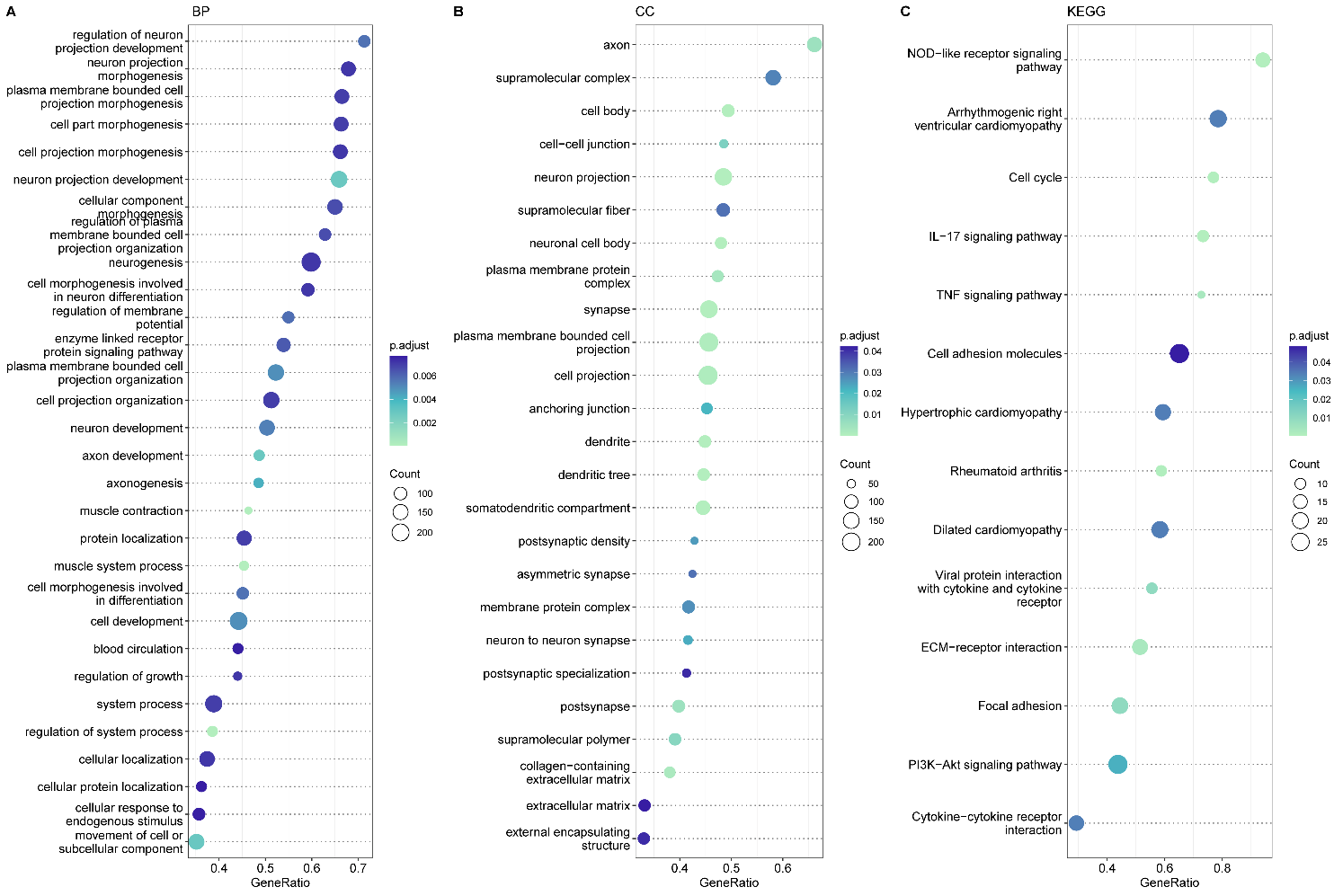


**Supplementary figure 4.** The differentially expressed genes between the two groups (IS1 + IS2 vs. IS4 + IS5) by GO (A, B) and KEGG pathway (C).
